# Measurement of Anticoagulation in Patients on Dabigatran, Rivaroxaban, and Apixaban Therapy by Novel Automated Thrombelastography

**DOI:** 10.1055/a-1692-1415

**Published:** 2021-11-09

**Authors:** Ramin Artang, Joao D. Dias, Mark Walsh, Kevin Bliden, Jorn D. Nielsen, Maren Anderson, Brian C. Thurston, Udaya S. Tantry, Jan Hartmann, Paul A. Gurbel

**Affiliations:** 1 Essentia Health St. Mary's Heart and Vascular Center, Duluth, Minnesota, United States; 2 Bispebjerg University of Copenhagen Hospital, Department of Cardiology, Copenhagen, Denmark; 3 Haemonetics Corp., Braintree, Massachusetts, United States; 4 Memorial Hospital of South Bend, Department of Energy Medicine, Sound Bend, Indiana, United States; 5 Sinai Center for Thrombosis Research and Drug Development, Sinai Hospital of Baltimore, Baltimore, Maryland, United States; 6 University of Minnesota School of Medicine, Duluth, Minnesota, United States; 7 Spartanburg Regional Medical Center, Division of Surgery, Spartanburg, South Carolina, United States

**Keywords:** clinical trials: oral anticoagulants, coagulation inhibitors, diagnosis management, apixaban, rivaroxaban, dabigatran

## Abstract

**Background** Direct-acting oral anticoagulants (DOACs) do not require monitoring. Measurement of DOAC effect would be useful in the event of bleeding, trauma, and thromboembolism while on anticoagulation. We evaluated the effectiveness of the investigational DOAC assays on the TEG®6s Hemostasis Analyzer to assess the anticoagulant effect of DOACs in patients treated for atrial fibrillation or deep vein thrombosis (DVT).

**Methods** Patients on treatment for a minimum of 7 days with standard doses of dabigatran, rivaroxaban, and apixaban were included. DOAC plasma concentrations and TEG®6s Reaction (R)-time were measured and correlated. The sensitivity, specificity, and negative predictive value (NPV) of R-time to detect DOAC concentrations of ≥30, ≥50, and ≥100 ng/mL were calculated.

**Results** A total of 189 patients were included, ( *n* = 50) on apixaban, ( *n* = 62) on rivaroxaban, ( *n* = 53) on dabigatran, and ( *n* = 24) on no DOAC were studied. Using the direct thrombin inhibitor (DTI) channel, R-time demonstrated strong linear correlation with dabigatran levels (r = 0.93, *p* < 0.0001). Using the antifactor Xa (AFXa) channel, R-time demonstrated strong nonlinear correlation with rivaroxaban and apixaban levels ( *r*_s_ = 0.92 and 0.84, respectively, *p* < 0.0001 for both). R-time revealed strong sensitivity and NPV in detecting low DOAC levels for the predefined concentrations.

**Conclusion** R-time measured by TEG®6s DOAC-specific cartridge has a strong correlation with concentrations of the most commonly used DOACs with high sensitivity and NPV for detecting lower drug levels that are considered clinically relevant for patients in need of antidote, or prior to urgent surgery. Further studies to determine the relation of R-time to clinical outcomes are warranted.

## Introduction

Laboratory assessment of the anticoagulant effect of the DOACs remains a challenge, a decade after this class of anticoagulants entered the market. While routine monitoring of these agents is not warranted, the ability to detect anticoagulation may be an important clinical tool in certain situations, such as overdose, bleeding, and urgent surgery. 1

The Thrombelastograph (TEG®6s) analyzer is a novel site-of-care test for global evaluation of hemostasis. The TEG®6s applies resonance-frequency viscoelasticity measurements and premixed disposable multichannel microfluidic cartridges. 2 The DOAC-specific cartridge for the TEG®6s system is an experimental prototype that was proven more sensitive and specific than the conventional Global Hemostasis cartridge in identifying the anticoagulant effect from DOAC therapy comparing DOAC treated patients to healthy volunteers without anticoagulation. 3 A study on healthy volunteers revealed a significant correlation between the TEG Reaction time (R-time) and blood DOAC levels using this cartridge. 4 In a recent study, the normal reference ranges for the DOAC-specific cartridge, and its effectiveness in detecting and classifying the DOAC treatment were established using blood samples from 160 healthy patients and 190 patients on treatment for atrial fibrillation and venous thromboembolism. 5 The clinically relevant DOAC concentration cut-offs, based on current available literature, are 30 ng/mL for urgent invasive procedures with high bleeding risk, 50 ng/mL for antidote administration, 6 7 and 100 ng/mL for thrombolysis in stroke. 8 The purpose of this present study was to demonstrate the correlation between the R-time and the DOAC plasma concentrations, as well as to assess sensitivity, specificity, and negative predictive value (NPV) of the R-time for the aforementioned clinically useful DOAC concentrations in the same cohort of patients.

## Materials and Methods

This study was conducted at the following five clinical sites in the United States from August 2016 until September 2017: Essentia Institute of Rural Health, Duluth, Minnesota, United States; Memorial Hospital of South Bend, South Bend, Indiana, United States; Spartanburg Regional Medical Center, Spartanburg, South Carolina, United States; Inova Heart and Vascular Institute, Falls Church, Virginia; and Inova Cardiology Ambulatory Research Center, Manassas, Virginia, United States. In accordance with the principles of Good Clinical Practice and the Declaration of Helsinki, Institutional Review Boards at each participating site conferred ethical approval. All participants were 18 years or older and gave informed consent prior to the enrollment.

For the on-DOAC group, patients were included if they were 18 years or older, and on a DOAC at doses recommended by the manufacturers for treatment of atrial fibrillation, venous thromboembolism, or thromboembolism prophylaxis for minimum of 7 days uninterrupted. The exclusion criteria were genetic bleeding disorders, known, or subsequently discovered inherited defects of coagulation (e.g., hemophilia or the von Willebrand disease), DOAC dosage outside of manufacturer's recommended range (e.g., study subject with renal impairment and supratherapeutic dose), heparin or low molecular weight heparin (LMWH) administered within 7 days prior to blood draw, on any other type on Food and Drug Administration (FDA) approved or experimental anticoagulant, bruising, wounds, or scarring in the area of venipuncture. Treatment with aspirin was not an exclusion criteria.

Blood samples were collected at a random time point at patient presentation to outpatient clinic or during inpatient presentation. A non-DOAC control group of patients was included in the analysis for comparison. Patients were included if they were 18 years or older. The exclusion criteria for the non-DOAC group were medical evidence of atrial fibrillation, deep vein thrombosis, or pulmonary embolism requiring anticoagulation, genetic bleeding disorders, known or subsequently discovered inherited defects of coagulation (e.g., hemophilia or the von Willebrand disease) on any medication containing heparin or LMWH within 7 days, a DOAC or other anticoagulant, any medications known to affect coagulation status, strict vegan diet, bruising, wounds, or scarring in the area of venipuncture.

### Blood Sampling and Analysis

Up to approximately 20 mL of blood was drawn standard venipuncture from each patient. No restrictions were made on use of tourniquet. The first 3 to 6 mL nonadditive tube was discarded at each draw. The second tube of 4.5 mL was collected in a standard 3.2% sodium citrate tube (BD Vacutainer, Franklin Lakes, New Jersey, United States), and was used for the TEG®6s analysis. The third tube of 4.5 mL was collected in a standard 3.2% sodium citrate tube and then centrifuged at 2,000 g for 10 minutes. The plasma samples were stored at −80°C for the measurement of DOAC concentrations. DOAC concentrations were measured by High Performance Liquid Chromatography/Tandem Mass Spectrometry (LC-MS/MS) assay at Quest Diagnostics (Wood Dale, Illinois, United States). 9

### Thrombelastography

The Thrombelastograph ®6s (TEG®6s, Haemonetics Corp, Boston, Massachusetts, United States) technique has previously been described in detail. 2 3 The TEG®6s system measures the clot viscoelasticity using resonance-frequency. The blood sample suspended in a microring structure within the cartridge is exposed to a fixed vibration frequency. With LED illumination, a detector measures vertical motion of the blood film. The frequency leading to resonance is identified and then converted to the TEG®6 system readout. The DOAC-specific cartridge contained kaolin in channel 1 (the citrated kaolin [CK] channel), ecarin in channel 2 (the direct thrombin inhibitor [DTI] channel), factor Xa (FXa) in channel 3 (the anti-FXa [AFXa] channel), and kaolin with abciximab in channel 4 (the functional fibrinogen channel). Based on unpublished pilot studies on patients treated with warfarin, the R-time was not altered beyond the established reference range for both DTI and AFXa channels with INRs ranging between 1.2 and 3.5. Heparin increased the R-time in the AFXa channel in a dose-dependent fashion but caused no change of R-time in the DTI channel. Experience with LMWH is pending and is considered as necessary part of the product development, but we anticipate similar response to the R-time on the AFXa and DTI channels as heparin given its effect on FXa inhibition. The DOAC specific cartridge is an experimental prototype that is currently not commercially available and is under investigation for use in patients who are treated with DOACs. The current protocol for the TEG®6s system is to allow the samples to stabilize for 10 minutes prior to analysis, unless the analysis is needed urgently for medical emergency. In the present study, all the R-time analyses were performed between 10 minutes and 2 hours after the phlebotomy.

### Statistical Analysis

The importance of measuring the R-time in patients treated with DOACs has been previously demonstrated. 3 4 We determined the correlation between R and DOAC concentrations. Pearson's correlation coefficient *r* was calculated for linear association between R-times and DOAC concentration. Spearman's rank correlation *r _s_* was calculated if the association of R-times and DOAC concentrations was nonlinear. Correlation coefficient values of >0.8 were considered strong. A *p* -value of <0.05 was considered statistically significant. If the association pattern did not fit a linear model, the best fit nonlinear model was selected. Goodness of fit expressed by *R ^2^* was calculated for both linear and nonlinear regressions. Concentrations of rivaroxaban, apixaban, and dabigatran were classified into three categories as follows: (1) <30 versus ≥ 30, (2) <50 versus ≥50, and (3) <100 versus ≥100 ng/mL. Logistic regression models compared R-times to the binary concentrations of (≥30, ≥50, and ≥100 ng/mL) to identify initial cut-off points to initiate the detailed analysis of sensitivity, specificity, positive predictive value (PPV), negative predictive value (NPV), positive likelihood (LR + ), and negative likelihood ratios (LR − ). Analysis of sensitivity, specificity, PPV, NPV, LR + , and LR− began at the R-time cut-off point identified by the logistic regression and continued for the next six R-times, consecutively. The best model fit was identified as the model with the highest LR+ and lowest LR− levels. 10 The rational for the sample size was previously described. 5 The correlations and graphs were generated using Prism software version 9 (GraphPad SoftwInc., La Jolla, California, United States). The sensitivity data were analyzed using IBM SPSS statistics (Version 23) predictive analytics software (IBM Corp. Armonk, New York, United States Released 2015).

## Results

**Fig. 1:**
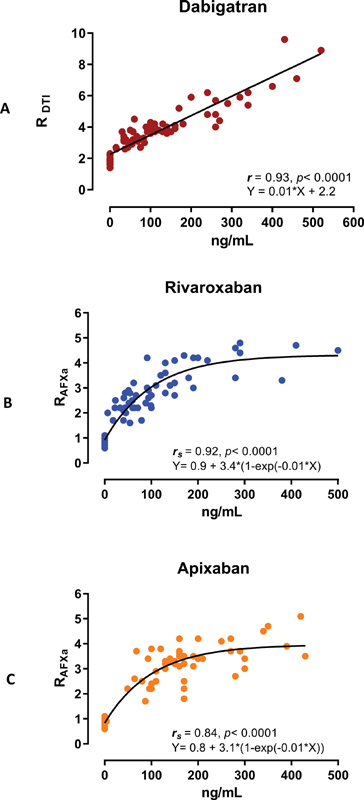
( **A** – **C** ) The scatter diagram of DOAC concentrations against R-time. R _DTI_ indicates using the DTI channel; R _FXa_ indicates using the AFXa channel. AFXa, antifactor-Xa; DOAC, direct-acting oral anticoagulants; DTI, direct thrombin inhibitor; R, reaction time.

A total of 189 patients were included in the study. Of them, 165 were in the on DOAC group and 24 patients were included on the non-DOAC group. The clinical characteristics for each DOAC and non-DOAC groups are outlined in Table 1 . Among the patients on DOAC, 50 were on apixaban, 53 on rivaroxaban, and 62 on dabigatran. The mean age of DOAC cohort was 71 ± 10.2 years and 42% were female. Among patients not anticoagulated, the mean age was 48 ± 11.6 years and 67% were female. Using the DTI channel, R-time demonstrated a strong linear correlation with dabigatran levels ( *r* = 0.93, *p* < 0.0001, and *R ^2^* = 0.86 for goodness of fit). Using the AFXa channel, R-time demonstrated strong nonlinear correlation with rivaroxaban and apixaban levels ( *r _s_* = 0.92 for rivaroxaban, *r _s_* = 0.84 for apixaban, *p* < 0.0001, and *R ^2^* = 0.86 for goodness of fit for both; Fig. 1 ).

**Table 1:** Subjects clinical characteristics

	Apixaban	Dabigatran	Rivaroxaban	Non-DOAC
	*n* = 50	*n* = 62	*n* = 53	*n* = 24
Mean age in years (SD)	72 (10.3)	70.9 (9.1)	70.8 (11.9)	48 (11.6)
Gender *n* (%)				
Female	25 (50)	24 (39)	25 (47)	16 (67)
Male	25 (50)	38 (61)	28 (53)	8 (33)
Ethnicity *n* (%)				
Caucasian	44 (88)	59 (95)	49 (92)	21 (88)
Other	6 (12)	3 (5)	4 (8)	3 (12)
DOAC indication *n* (%)				
Atrial fibrillation or flutter	43 (86)	62 (100)	45 (85)	
DVT/PE treatment	4 (8)	0	6 (11)	
DVT prophylactic	3 (6)	0	2 (4)	
Comorbidities *n* (%)				
Hyperlipidemia	27(54)	37 (60)	24 (45)	5 (21)
Hypertension	38 (76)	43 (69)	33 (62)	10 (42)
Heart failure	14 (28)	7 (11)	6 (11)	
Diabetes	12 (24)	16 (26)	8 (15)	
Hypothyroidism	4 (8)	8 (13)	6 (11)	1 (4)
Coronary artery disease	3 (6)	6 (10)	10 (19)	
Antiplatelet therapy ConMed *n* (%)				
Aspirin	9 (18)	17 (27)	10 (19)	2 (8)
Clopidogrel	4 (8)	1 (2)	1 (2)	
Prasugrel			1 (2)	

Abbreviations: DOAC, direct-acting oral anticoagulants; DVT, deep vein thrombosis; PE, pulmonary embolism; SD, standard deviation.

The DOAC levels among the non-DOAC group were 0. Among the 50 patients on apixaban, one patient had a levels of 29 ng/mL or less, one patient between 30 and 49 ng/mL, and six patients between 50 and 99 ng/mL. Among the 53 patients on rivaroxaban, 4 had a level of 29 ng/mL or less, 7 had between 30 and 49 ng/mL, and 17 had between 50 and 99 ng/mL. Among the 62 patients on dabigatran, 2 had a level of 29 ng/mL or less, 9 had between 30 and 49 ng/mL, and 17 had between 50 and 99 ng/mL. The sensitivity, specificity, LR + , LR − , and NPVs for the R-time to detect DOAC concentrations of ≥30, ≥50, and ≥100 ng/mL for the entire cohort of on-DOAC and non-DOAC patients are presented in Table 2 . R-time revealed strong sensitivity and NPV for the predefined DOAC concentrations. Due to only single-acquired concentration between 30 and 49 ng/mL in the apixaban group, no sensitivity and specificity calculations were performed in that range.

**Table 2:** Sensitivity, specificity and negative predictive value of R-time to detect DOAC concentrations above thresholds mentioned

DOAC	Threshold	R-time	Sensitivity	Specificity	LR+	LR−	**NPV**
	≥(ng/mL)	≥(min)	%	%			**%**
Dabigatran							
	30	2.6	100	92 (82–100)	13	0	100
	50	3.1	94 (88–100)	83 (70–95)	5.5	0.07	91 (81–100)
	100	3.4	100	82 (72–93)	5.6	0	100
Rivaroxaban							
	30	1.7	98 (94–100)	86 (73–99)	6.9	0.02	96 (88–100)
	50	2.1	95 (89–100)	80 (67–93)	4.8	0.06	93 (84–100)
	100	2.6	96 (78.9–99.9)	85 (72.4–93.3)	5.1	0.05	98 (93–100)
Apixaban							
	30	c					
	50	1.7	100	96 (88–100)	25	0	100
	100	2.2	98 (93–100)	81 (67–95)	5	0.03	96 (89–100)

Abbreviations: AFXa, antifactor-Xa; AUC, area under the curve; DOAC, direct oral anticoagulant; DTI, direct thrombin inhibitor; LR + , positive likelihood ratio; LR − , negative likelihood ratio; NPV, negative predictive value; R, reaction time.

Note: The reference range of R-time for the FXa channel is 0.6–1.5 minutes, and for DTI channel is 1.6–2.5 minutes.

a Indicates using the DTI channel.

b Indicates using the AFXa channel.

c Due to only single acquired concentration between 30 and 49 ng/mL, no calculations were performed.

## Discussion

To date, this is the largest study correlating the DOAC concentrations to TEG-R value using the DOAC-specific cartridge with TEG®6s device. In this study, a strong correlation between R-time and DOAC concentration in 165 patients on DOAC was demonstrated. The R-time also revealed high sensitivity and NPV for the low doses of the three most commonly used DOAC agents.

This technology may be useful in clinical situations requiring rapid assessment of the coagulation status in patients who present with acute bleeding while on DOAC treatment. Other clinical situations where such test may prove to be useful are patients on DOAC needing urgent surgery, and prior to DC-cardioversion, or radiofrequency ablation for patients with atrial fibrillation when there is uncertainty about the hemostatic function or adequacy of anticoagulation. The R-time assessment test duration was under 6 minutes.

The association observed between R-time, as well as the concentration of the FXa inhibitors rivaroxaban and apixaban, was best fitted in a nonlinear pattern, as increasing concentrations of the FXa inhibitor concentrations would not cause further increase of the R-time displaying a plateau in the curve ( Fig. 1B, C ). We hypothesize that this observation may be related to the fixed concentration of FXa used in the FXa channel chamber. Alternative explanation may be the fixed amount of FXa in the whole blood sample that is present in the chamber, thus limited number of FXa binding sites for the FXa inhibitor molecules. It is therefore plausible that at higher concentrations of FXa inhibitor (>150–200 ng/mL), a saturation point may be achieved where higher levels of the in vivo FXa inhibitor concentration would no longer impact the effect of FXa in converting prothrombin to thrombin and thereby fibrin generation. Since the R-time reflects the beginning of fibrin–platelet clot generation, the mechanism described above may explain the plateau in R-time with further increase of the FXa inhibitor concentration in the present study. This hypothesis requires further investigation with escalating FXa dose in in vitro samples analyzed. The correlation of dabigatran concentrations and R-time was linear in the concentrations observed in this study.

The routine monitoring of patients on chronic DOAC treatment, without thrombotic or bleeding events, is not recommended by the guidelines. 11 12 The challenge for the clinicians is how to respond to a measured level that falls at the upper or lower end of a very wide range reported in patients on chronic DOAC treatment. 13 Secondary analysis of the randomized DOAC trials has revealed association of higher drug concentration with bleeding and lower concentrations with thrombotic events. There is so far, no evidence that adjustment of the DOAC dose, beyond what was demonstrated in the original DOAC trials and stated in the prescription information, would improve the clinical outcome. Patients' propensity for thrombosis or bleeding may differ significantly based on patient characteristics including but not limited to age and renal function. It is therefore possible that the same concentration of DOAC in plasma can result in adequate protection in some patients but thrombosis or bleeding in others. 14 The global coagulation assay parameters, such as the R-time in Thrombelastography and lag-time in thrombin generation assay, have a statistically significant correlation with DOAC concentration levels but do not offer an exact prediction of the DOAC concentration. 4 15 The latter observation is further confirmed as seen by the scatter in Fig. 1 in the present study. Our observation reveals that the strength of the TEG®6s technique beyond its short turnaround time may be its high sensitivity and NPV for the predefined DOAC concentrations in a qualitative binary pattern (≥50 or 100 ng/mL of drug present vs. no clinically significant amount present) rather than quantitative (ng/mL) assessment of DOAC concentrations. For concentrations of 30 ng/mL or lower and for potential clinical application of this technology in general, these observations will need to be verified with much larger cohorts and more robust and consistent high sensitivity and NPVs across different predefined low concentrations. The present study was not designed to answer the question of clinical outcomes correlation with R-times but may be considered as hypothesis generating research. Establishing such correlation is therefore warranted using the results from large clinical studies, associating DOAC concentrations and different coagulation parameters, including the global assays with the clinical outcomes or conducting new studies where the global assays are included.

## Limitations

The limitation of the present study includes a lack of adequate data on edoxaban, the other currently FDA approved FXa inhibitor. Given only four patients included edoxaban group, no meaningful analysis could be performed on the data which were omitted from this manuscript. The thresholds achieved in the present study on rivaroxaban and apixaban can therefore not be used or deduced for edoxaban. The study design did not include hour-by-hour monitoring of the DOAC level but rather approaching the patients consecutively as they presented to the clinic or the hospital, regardless of the time of DOAC intake. The sensitivity and specificity calculations on apixaban for levels between 30 and 49 ng/mL were therefore less certain. A larger number of observations at DOAC levels less than 50 ng/mL would have further strengthen the sensitivity and specificity of this technique in lower concentrations where it considered clinically relevant. The study would furthermore have demonstrated much higher specificity, NPV, and LR+ if the non-DOAC cohort was larger by design providing a larger true negative value.

## Conclusion

In conclusion, R-time measured by TEG®6s DOAC-specific cartridge has a strong correlation with concentrations of the most commonly used DOACs. This technique has high sensitivity and NPV for detecting lower drug levels that are considered clinically relevant for potential use in patients in need of antidote or prior to urgent surgery. Further larger studies with focus on lower concentrations and the relation of R-time to clinical outcomes are warranted. This novel assay has promise in the personalization of antithrombotic therapy in patients with cardiovascular and thrombotic diseases.

## Notes

**What Is Known About This Topic?**

- Prior studies on the Thrombelastograph TEG®6s have shown favorable correlation between direct oral anticoagulants (DOACs) concentrations and TEG Reaction-time in small number of healthy volunteers.

**What Does This Paper Add?**

- We studied the correlation of TEG®6s Reaction (R)-time to DOAC plasma concentrations in 165 patients on chronic DOAC treatment.
- TEG®6s R-time demonstrated highly significant correlation with dabigatran, rivaroxaban, and apixaban blood concentrations.
- The R-time demonstrated high sensitivity and negative predictive value to detect DOAC concentrations of ≥30, ≥50, and ≥100 ng/mL.
